# Risk Factors of Extended-Spectrum β-Lactamase Producing Enterobacteriaceae Occurrence in Farms in Reunion, Madagascar and Mayotte Islands, 2016–2017

**DOI:** 10.3390/vetsci5010022

**Published:** 2018-02-23

**Authors:** Noellie Gay, Alexandre Leclaire, Morgane Laval, Guillaume Miltgen, Maël Jégo, Ramin Stéphane, Julien Jaubert, Olivier Belmonte, Eric Cardinale

**Affiliations:** 1 Animals, Health, Territories, Risks and Ecosystems, Avenue Agropolis, 34398 Montpellier CEDEX 5, France; laval.morgane1@gmail.com (M.L.); jego.mael@gmail.com (M.J.); ramin.stephane@hotmail.fr (R.S.); eric.cardinale@cirad.fr (E.C.); 2 Bacteriology laboratory, Félix Guyon Hospital, Saint-Denis, 97400 Reunion, France; olivier.belmonte@chu-reunion.fr; alexandre.leclaire@yahoo.fr (A.L.); guillaume.miltgen@chu-reunion.fr (G.M.); julien.jaubert@chu-reunion.fr (J.J.); olivier.belmonte@chu-reunion.fr (O.B.); 3 UMR PIMIT, CNRS 9192, INSERM U1187, IRD 249, F-97418 Sainte-Clotilde, La Réunion, France

**Keywords:** Indian ocean, livestock, extended-spectrum β-Lactamase producing Enterobacteriaceae, risk factors, CTX-M, enzymes

## Abstract

In South Western Indian ocean (IO), Extended-Spectrum β-Lactamase producing Enterobacteriaceae (ESBL-E) are a main public health issue. In livestock, ESBL-E burden was unknown. The aim of this study was estimating the prevalence of ESBL-E on commercial farms in Reunion, Mayotte and Madagascar and genes involved. Secondly, risk factors of ESBL-E occurrence in broiler, beef cattle and pig farms were explored. In 2016–2017, commercial farms were sampled using boot swabs and samples stored at 4 °C before microbiological analysis for phenotypical ESBL-E and gene characterization. A dichotomous questionnaire was performed. Prevalences observed in all production types and territories were high, except for beef cattle in Reunion, which differed significantly. The most common ESBL gene was *bla*_CTX-M-1_. Generalized linear models explaining ESBL-E occurrence varied between livestock production sectors and allowed identifying main protective (e.g., water quality control and detergent use for cleaning) and risk factors (e.g., recent antibiotic use, other farmers visiting the exploitation, pet presence). This study is the first to explore tools for antibiotic resistance management in IO farms. It provides interesting hypothesis to explore about antibiotic use in IO territories and ESBL-E transmission between pig, beef cattle and humans in Madagascar.

## 1. Introduction

Extended-spectrum β-Lactamase producing Enterobacteriaceae (ESBL-E) is a public and veterinary health burden worldwide and particularly in West Indian ocean countries [1]. These multi-resistant bacteria have been identified as a priority in terms of epidemiological surveillance in humans and animals from the Indian Ocean Commission (IOC) state members (i.e., Comoros, Madagascar, Mauritius, Reunion and Seychelles) and Mayotte (French oversea territory) [1].

ESBL-E are resistant to almost all beta-lactam antibiotic drugs including third generation cephalosporin (3GC), co-resistance is often observed with other classes of antibiotics such as fluoroquionolones, aminoglycosides, sulfonamides and tetracyclins, leading to the use of last-resort antibiotics (i.e., carbapenems) in ESBL-E infections in humans [2].

The occurrence of ESBL-E has been identified in broiler and swine farms in Europe [3,4,5] and the CTX-M β-lactamases is the most frequently detected enzyme in livestock, especially *bla*_CTX-M-1_ [4].

Selection pressure exerted by antibiotic drugs on microbiota favours carriage and persistence of ESBL-E in humans (hospital and community) [6,7], livestock and pets [7,8,9]; thus, all could act as potential reservoirs of ESBL-E.

The main known risk factor identified in ESBL-E occurrence in livestock was “use of 3GC or fourth generation cephalosporin (4GC) (ceftiofur, cefoperazone and cefquinome) in the last 12 months” in dairy and pig farms [10,11].

Other risk factors such as storage of slurry in a pit, operating an open herd policy and infrequent cleaning of calf feeding equipment were also identified in dairy farms [4] and fish ponds presence in poultry farms of Vietnam [12].

In IOC, no estimate of ESBL-E prevalence in livestock was available. Thus, the aim of this study was first estimate the prevalence of ESBL-E on beef cattle, broiler and pig commercial farms in Reunion, Mayotte and Madagascar Islands and identify ESBL enzymes occurrence in each production type and territory. Secondly, potential risk factors of ESBL-E occurrence in poultry, beef cattle and pig farms were explored.

## 2. Materials and Methods

### 2.1. Study Population

Reunion and Mayotte are French overseas territories located in South Western Indian ocean. Reunion with an area of 2512 km^2^ is home for around 850,996 people [13]. In Reunion, 156 poultry producers, 340 pig producers and 331 beef cattle producers are structured in official breeding organization and could be considered as intensive or partially free ranging [14].

Mayotte with an area of 374 km^2^ is home for around 235,132 people [13]. One hundred fifty modern poultry producers and 3600 beef cattle farms are recorded in this territory [15]. However, twenty poultry producers and 320 beef cattle producers are structured in breeding official organizations [16]. 

Madagascar is the fifth largest island in the world, with a land mass of 587,000 km^2^ and 24.24 million inhabitants in 2016 [17]. Its economy is based essentially on agriculture and tourism; producer census was not available at the Direction of Veterinary Services of the Ministry of Livestock Production [18]. 

### 2.2. Sampling

From February to August 2016, broiler, pigs and beef cattle farms were sampled in Reunion. Due to a foot-and-mouth outbreak in Mauritius Island, sampling had to be stopped in beef cattle in Reunion for sanitary reasons. Sampling was reported to August 2017 for beef cattle. In Mayotte, beef cattle and broiler were sampled from September to October 2016, no pig farms were present in this territory due to mostly Muslim community representation; thus, no sample of pigs was collected. In Madagascar, sampling was performed in November 2016. Beef cattle were sampled in Antsirabe, broiler in Mahitsy and pig farms in Imerintsiatosika, known to be key production sites. It is to be noted that broiler and beef cattle farms from Mayotte and Madagascar could also raise few hen and dairy cattle in the farm without being the main commercial activity.

In each territory, the sample size of thirty breeding farms of each livestock production sector were targeted. Samples were collected using boot swabs Sterisox^®^. Number of samples depended on the house’s surface area, one Sterisox^®^ covered 100 m² of building. If possible all boxes were visited and livestock gathering points (e.g., water pond, watering trough) were also sampled. Number of samples per farm varied between one and five.

All samples were immediately maintained at 4 °C before analyses proceeded within 48 h after reception (transport within the day for Reunion and within one week for Mayotte and Madagascar).

No ethical approval was needed as non-invasive sampling methods were used to identify farm ESBL-E sanitary status.

### 2.3. Laboratory Investigations

#### 2.3.1. ESBL-E Phenotype

Sterisox^®^ boot swabs were incubated 20 ± 4 h at room temperature with 100 ml of physiological water and 900 µL of Brain-Heart Infusion broth (BioMérieux SA, Marcy l’Etoile, France). Ten µL of the enriched suspension was directly streaked onto selective chromogenic agar plates (ChromID-ESBL, Biomérieux, Marcy l’Etoile, France) and incubated overnight at 37 °C under aerobic condition. Presumptive ESBL-producers were sub-cultured individually on Drigalski lactose agar and bacterial species identification performed using MALDI-TOF mass spectrometry (Bruker Daltonics, Breme, Germany). All Enterobacteriacae isolates identified, one or more by positive farms, were considered ESBL-E if confirmed by the combination disc test according to the European Committee on Antimicrobial Susceptibility Testing guidelines [19]. Thus, Muller Hinton agar with cefotaxime, ceftazidime, cefixime and cefepime disks with and without clavulanic acid allowed testing. The result was considered positive if the inhibition zone diameter was ≥5 mm larger with clavulanic acid than without for at least on cephalosporin tested.

If ESBL-E were identified, antibiograms were performed on isolates with ertapenem (ETP), nalidixic acid (NA), ofloxacin (OFL), gentamicin (GEN), Amikacin (AMK), trimethoprim/sulfamethoxazole (SXT) and tetracycline (TCN) tested.

#### 2.3.2. Characterization of ESBL Genes

ESBL-producing isolates were randomly selected per livestock production sector for each territory (except Reunion with 35 *E. coli* isolates). Total DNA was extracted using the NucliSens® Easymag^®^ system (Biomérieux, Marcy l’Etoile, France) according to the manufacturer’s instructions. Extracted eluates were stored at −80 °C. Molecular characterization was performed using Check-MDR CT103XL array test (Check-Points Health B.V., Wageningen, Netherlands) for identification of ESBL genes (i.e., encoding BEL, CTX-M-1, CTX-M-2, CTX-M-9, CTX-M-8/25, GES-ESBL, PER, SHV-ESBL, TEM-ESBL, VEB) and discriminated ESBL and non-ESBL TEM and SHV variants. The assay consisted in a two-step amplification process of the ESBL target sequences, followed by a colorimetric microarray detection of the reaction products. Image analysis and interpretation were provided by Check-Points “5-2-2015” software (Check-Points Health B.V., Wageningen, The Netherlands).

### 2.4. Questionnaire

A dichotomous questionnaire to assess potential risk factors on farms was developed. Data regarding farm building, biosecurity measures, breeding practices including management of knackery, water quality, quarantine and effluent, vector control, cleaning and disinfection techniques, use of antibiotics and questions related to the breed like housing system and origins of animals were collected (See questionnaire annex). Answers were cross-checked by direct observation and corrected if necessary.

### 2.5. Risk Factors Analyses

A farm was considered positive if at least one boot swab was found positive for ESBL-E in bacteriological analysis. A farm was considered negative if all boot swabs samples were negative for ESBL-E.

Explanatory variables considered for analysis were categorical. If fewer than five observations recorded in a category the variable was excluded. The variable to be explained was ESBL-E occurrence in the livestock production sector in each territory. Bivariate analyses were performed using Fisher test (*p* < 0.05).

For generalized linear models (GLM), a preliminary step aimed at evaluating association between explicative variables and ESBL-E farm status with bivariate analyses in each livestock production sectors (including all three territories). Factors associated with ESBL-E positivity with a *p*-value < 0.20 were offered to a full model form multivariate analysis (GLM). The variable territory was not included in models as it was significantly associated with other variables. Interactions between variables were not including in the models. The preferred model was the one with the minimum Akaike information criterion (AIC). Goodness of fit test were also performed. R software (R Development Core team, 2012) was used to perform statistical analysis (https://www.r-project.org/).

## 3. Results

### 3.1. Prevalence Observed, Bacterial Diversity and Antibiogram Results

In Reunion, high prevalences were observed in poultry (70.0% ± 16.7%) and pig farms (53.3% ± 18.2) (Table 1). Prevalence differed significantly between livestock production type in Reunion (*p*-value < 0.001) with a low prevalence observed in beef cattle farms (3.7% ± 5.1%). In Mayotte and Madagascar, no difference in prevalence was observed between livestock type in each territory (*p*-value > 0.05).

Comparing prevalence among poultry production in the three territories, no difference was observed (*p*-value = 0.94). In pig production, the prevalence differed significantly between Madagascar and Reunion (*p*-value < 0.005). Finally, in beef cattle the prevalence between the three territories differed significantly (*p*-value < 0.001).

In Reunion, four different species were found among Enterobacteriacae isolates with two species (*Escherichia coli* and *Enterobacter cloacae complex*) in both poultry and beef cattle farms, three species in pig (*E. coli*, *Klebsiella pneumonia* and *Citrobacter freundii*) (Table 2).

In Mayotte, Enterobacteriacae diversity was reduced to *E. coli* and *E. cloacae* complex in both poultry and beef cattle production.

In Madagascar, an important diversity of species was found among Enterobacteriacae isolates with six different species identified in all types of production. Species diversity varied according to the production type with five species identified in pig production, three in beef cattle and poultry production.

The main represented species in all territories and all types of production was *E. coli* with 89.0% (n = 307) of all Enterobacteriaceae isolates (N = 345), 95.1% (n = 292) out of them being ESBL producers (Table 2).

No phenotypic resistance to ertapenem (ETP) was identified in ESBL-E isolates (Table 3). Resistance to nalidixic acid (NA) was high in ESBL producing *E. coli* in beef cattle from Reunion (50.0%) and in Madagascar both in poultry (28.6%) and pig (25.0%) farms. Resistance to ofloxacin (OFX) was high in ESBL producing *E. coli* in pig production both in Madagascar (21.4%) and Reunion (25.0%). Resistance to gentamicin (GEN) was elevated in ESBL producing *K. pneumoniae* in Madagascar. No resistant profile to amikacin (AKN) was identified in all territories. In ESBL producing *E. coli* trimethoprime/sulfamethoxazole (SXT) resistance was high in Reunion both in poultry and pig production (75.0% and 87.5% respectively). ESBL producing *E. coli* most resistant profiles to tetracycline (TE) were observed in Madagascar (i.e., 92.9% in broiler, 75.0% in pigs and 50.0% in beef cattle).

### 3.2. ESBL Identification

ESBL-producing isolates were randomly selected per livestock production sector for each territory, except for Poultry in Reunion. The most common ESBL gene identified in all territories and production type was *bla*_CTX-M-1_ which accounted for 53.7% (n = 49) of all *E. coli* isolates tested (N = 95), followed by *bla*_CTX-M-15_ (29.5%, n = 28) (Table 4). The higher diversity in ESBL gene was found in poultry production from all territories.

### 3.3. Explanatory Factors of ESBL-E Occurrence in Livestock Sectors Production in Reunion, Madagascar and Mayotte, 2016–2017

Univariate Odds Ratios (ORs) for the occurrence of ESBL-E in each livestock production sectors and territory are presented in (Table 5). Premises building constructed after 1999 were associated with an increased probability of ESBL-E occurrence in broiler production in Reunion. In pig production, changing shoes/boots before entering the building was associated with an increase of ESBL-E occurrence whereas rodent control by a company and two disinfections between two consecutive batches of fattening pigs were associated with a decreased probability of ESBL-E occurrence.

In Madagascar, absence of chick introduction in the farm (self-production) in broiler farms was associated with decreased ESBL-E occurrence. Clearing space around the farm was associated with a decreased probability of ESBL-E occurrence in beef cattle production.

Generalized linear models explaining ESBL-E occurrence (all territories included) varied between livestock production (Table 6). In broiler, “water quality control” was identified was associated with decreased of ESBL-E occurrence (OR: 0.12); the best model selected the variables “distance to another farm”, “foot bath at entrance,” “water quality” and “water storage tank” (AIC: 93.98).

In pig production, “other farmers visiting the farm,” “soak the floor,” “detergent use for cleaning” and “antibiotic use recently” were identified in the best model (AIC: 65.09).

For beef cattle, the best model kept “livestock size,” “antibiotic use,” “disinfestation,” “clearing space around the building,” “pet presence” and “water storage tank” (AIC: 83.53).

## 4. Discussion

Our study pointed out high ESBL-E prevalence in Madagascar, Reunion and Mayotte livestock commercial farms. Overall ESBL genes diversity in *E. coli* was reduced with *bla*_CTX-M-1_ mainly identified. In Madagascar, all genes identified in pig and beef cattle were *bla*_CTX-M-15_, main enzyme observed in humans [20,21]. It could confirm circulation of ESBL-E between human and livestock. Concrete factors associated with an increased risk of ESBL-E occurrence in farms were identified such as pet presence, farmer visits and recent antibiotic use. Finally, biosecurity and hygienic measures (e.g., disinfection, water quality control, detergent use) were globally reducing ESBL-E occurrence in IOC farms.

Our study clearly pointed a high ESBL-E prevalence in Madagascar, Reunion (except beef cattle) and Mayotte. Prevalence estimate was not accurate as obtained with a limited sample size; Madagascar ESBL-E prevalence calculated could neither estimate the overall prevalence in this large territory nor be the reflect of livestock farms diversity. If ESBL characterization allowed, for the first time, to identify a circulation of *bla*_CTX-M-1_ in all livestock types, the limited number of ESBL found in each livestock and IO territory (N = 10) cannot rule out patterns. Less diversity was expected by livestock type (e.g., in poultry in each territory, pigs from Reunion and beef cattle from Mayotte) and could highlight needs of further enzyme identification as diversity could not be captured as a whole.

No phenotypic resistance to ertapenem in ESBL-E isolates was identified, which is in accordance with the absence of carbapenemase producing Enterobacteriacae (CPE) detection in IO livestock in 2018 [1]. However, use of CPE selective media would be more suitable for CPE detection. Resistance to fluoroquinolone could be low in Mayotte as no resistance to ofloxacin was observed but should be confirmed as few isolates were tested. Hypothesis about risk factors identification in our study was opportunistic and case control or cohort study designs to rule out ESBL-E control measures would be needed. Furthermore, antibiotic drug use recently was identified as increasing ESBL-E occurrence in IO farms but the farmers were not able to tell which antibiotic drug was used. Further studies should be undertaken to evaluate antibiotic drugs consumption and practices in IO farms.

In broiler production, the estimated prevalence in IO territories was higher than 50.0% reported in 2012 in Germany [22] but similar to 70.0% reported in Japan in 2007 [23]. In India, in 2014, among 87.0% of ESBL-E were detected in broiler and 42.0% in layer farms [24]. In pig farms, the prevalence in IO was higher than 8.3% reported in pigs in Japan in 2007 [23]. For Madagascar, it was similar to the 88.2% of ESBL-E positive farms observed in 2012 in Germany [22]. ESBL-E occurrence of Mayotte and Madagascar beef cattle farms were similar to data reported from other studies in Germany with 73.3% of farms tested positive in 2011 to 2012 (Bavaria) [25] and 54.4% in 2012 in Mecklenburg-Vorpommern [22]. In Reunion, the prevalence of ESBL-E in beef cattle farms tends to be significantly lower than in other territories. It could reflect the effectiveness of the French governmental antibiotic reduction plan (Ecoantibio) in Reunion and better biosecurity. Mayotte is a French oversea territory, breeding practices are clearly different from Reunion with mixed livestock farms and could explain observed differences.

Finally, the high ESBL-E prevalence observed in IO territories could point to important antibiotic drug use and/or misuse, including cephalosporins. This is particularly true for pigs in Madagascar where high antibiotic residues were reported in pork products at abattoirs [26]. 

Main ESBL-E co-resistance were observed in Madagascar (i.e., ofloxacin, tetracyclin, nalidixic acid and gentamicin) and Reunion (i.e., ofloxacin, nalidixic acid and trimethoprime/sulfamethoxazole). High ESBL-E co-resistance observed in Madagascar could point out a drug overuse, particularly for widely available oral agents [1]. Nalidixic acid resistant isolates were resistant to ofloxacin in Reunion and Madagascar pig productions as observed in majority of cases [27]. Fluoroquinolone resistance was high in ESBL producing *E. coli* in pig production of both territories which could indicate past or present use/misuse of this critically important antimicrobial drug. Pig production was identified as the most important antibiotic consumer worldwide [28]. French national data indicated that fluoroquinolones use was higher in cattle production than in pig and poultry production [29]; trends, not estimated in IO French overseas territories, could differ from mainland France. 

The most common ESBL gene identified in *E. coli* isolates tested was *bla*_CTX-M-1_ (54.4%) as observed in food-producing animals in European countries [30]. CTX-M β-lactamase is largely located on plasmids, which allows the horizontal transfer between Enterobacteriaceae [31] and explains the current epidemic spread of this enzyme worldwide. 

Overall ESBL gene diversity was reduced in our study with circulation of few genes by production type (e.g., *bla*_CTX-M-1_ in pig and poultry from Reunion and *bla*_CTX-M-15_ in pigs and beef cattle in Madagascar). It probably indicated a common past source of contamination with introduction of ESBL-E carriers and diffusion due to close contact in livestock as reported with *bla*_CTX-M-14_ in cattle from the United Kingdom [10]. Thus, overall introduction/exchanges of ESBL-E between reservoirs and environment seems limited as observed by Dorado-Garcia in the Netherlands (2005–2015) [32]. A more diverse ESBL genes pool was identified in IO poultry production with at least three different genes detected in each territory. Most of ESBL genes were *bla*_CTX-M-1_ but SHV-ESBL and TEM-ESBL genes were also identified as in Dutch broilers [33]. This diversity of ESBL genes in poultry could be related to close contact with poultry house surrounding environment. Interestingly, *bla*_CTX-M-15_ was observed in pig production, beef cattle and poultry from Mayotte and Madagascar; It is the main enzyme observed in humans in IO [1,20,21] and circulation of ESBL-E between human and livestock could be suspected.

In broiler farms, “Premises building constructed after 1999” and “change of shoes/boots before entering the building” were significantly increasing ESBL-E occurrence in Reunion. Both factors were difficult to explain as related to improved biosecurity measures. Antibiotic drug use could be higher in modern farms and “change of shoes/boots” was identified also as a risk factor ESBL-E occurrence in Vietnam poultry production [12] confirming that further investigations are needed to identify a potential confounding explanatory factor. In Madagascar, “chick production in the farm” significantly reduced occurrence of ESBL-E. This is in accordance with a vertical ESBL-E transmission into the production chain through external introduction such as imported day-old grandparent chickens as in Dutch poultry farms [34]. In all IO territories, “water quality control” was a protective factor of ESBL-E occurrence in commercial farms. It was in accordance with studies on *Campylobacter* spp. that showed that electrolyzed water or chlorinated-water allowed reducing bacterial presence [35,36]. Rural surface water may become a large reservoir of antibiotic residues and resistant bacteria [37], thus, in order to minimize transmission of enteropathogens, drinking water should be of potable quality to ensure freedom from enteric pathogens [37]. 

In pig farms, both “rodent control” and “two disinfections between two consecutive batches” were significantly reducing ESBL-E occurrence in Reunion. Both measures are related to biosecurity and hygiene helping to control disease and antibiotic resistance spread. In all IO territories, “recent antibiotic use”, “soak the floor” and “farmer visits” were associated with an increase of ESBL-E occurrence in pig production whereas “detergent use for cleaning” was associated with a decreased occurrence. ESBL-E occurrence could be more determined by the presence or absence of cephalosporin use at the farm as in Dutch pig production [38]. “Others farmer visits” has never been identified as increasing ESBL-E occurrence and could be more related to the frequency of visits as observed with the veterinarian in cattle farms in Israel [39]. Visitors could contribute to ESBL-E introduction and could carry/share material that favours transmission pathways. Detergent use for cleaning was associated with a decreased ESBL-E occurrence in IO pig production. Using effective detergent for cleaning was identified to decrease the risk of batch infection by Enterobacteriaceae such as *Salmonella* sp. [40]. However, “soak floor” practice in IO pig farm production could be explained by wrong biosecurity practices; for instance, let water for a too short period could not allow complete cleaning. For instance, a period of one-hour soak time may could be insufficient to demonstrate a significant difference in organic matter removal in pig pens [41]. Thus, cleaning and disinfection processes are a cornerstone in ESBL-E eradication which was obtained in pig farms under specific disinfection procedures [42].

In beef cattle production, “clearing space around the building “and “clean condition around the farm” reduced significantly ESBL-E occurrence in Madagascar. This explanatory variable could be related to a confounding factor; garbage presence in the farm probably attracting potential ESBL-E reservoirs such as dogs, cats or rodents. Accordingly, pet presence in the farm was identified as increasing ESBL-E occurrence in IO beef cattle farms. This finding was in accordance with Santman-Berends et al. 2017 [43] which found cat presence as an explanatory factor of ESBL-E occurrence in organic herds in the Netherlands in 2011. It could be due to the fact that pets could be both given antibiotic drugs by owners and/or play a role of reservoir/vector of ESBL-E from the close environment. Furthermore, “recent antibiotic use” was associated with an increased ESBL-E occurrence in beef cattle farms. However, 3rd or 4th generation cephalosporin use in IO beef cattle farm was not studied while use was estimated to increase by nearly 4 times ESBL producing *E. coli* in dairy farms if used in the last 12 months [10].

Factors associated with a decrease of ESBL-E occurrence in IO beef cattle farms were “livestock size” and “disinfection”. IO big farms, herd size (>25 cattle), could apply stricter biosecurity measures. However, Adler et al. (2017) reported that an increased density was associated with more ESBL-E carriage in Israeli cattle farms [39]. As discussed before, cleaning and disinfection seems to be cornerstones in ESBL-E management and hygiene paucity was identified as a risk factors of ESBL-E occurrence on dairy farms (e.g., storage of slurry in a pit, infrequent cleaning of feeding equipment) [10].

In IO ESBL-E occurrence in 2016–2017 was high probably pointing out antibiotic drug overuses and/or misuses and particularly cephalosporins. The situation could be reversible if better practices were implemented regarding antibiotic use. For instance, in the Netherlands in 2010–2011, 20% of prevalence was observed if no cephalosporin was used (3CG and 4CG) within the preceding year in pig farms and 79% if those antibiotics were used [11]. 

*Bla*_CTX-M-15_ gene, mainly identified in humans both in hospital and community, was observed in IO livestock and particularly Madagascar Further investigations, including complete genome sequencing, are needed to evaluate the hypothesis of ESBL-E transmission and diffusion between reservoirs in this territory. Finally, interesting factors related to biosecurity and hygiene measures in commercial farms were identified (e.g., controlled water, disinfection, rodent control) to control ESBL-E occurrence.

## 5. Conclusions

Finally, this study in IOC commercial farms pointed out high ESBL-E prevalences in livestock, except beef cattle in Reunion. It highlighted probable antibiotic overuse/misuse in farms contributing in ESBL-E selection. It confirmed the need to evaluate consumption and use of antibiotic drugs in IOC territories. Concrete protective and risk factors of ESBL-E occurrence (e.g., pet presence, detergent use for cleaning) were identified, even if further investigations are needed to reinforce these results. This study is the first to explore the situation of antibiotic drug resistance in farm animals and explore potential tools for management of ESBL-E in IO farms. As a whole, it confirms the need for improving in biosecurity and hygienic measures as efficient means to reduce antibiotic resistance in livestock. Finally, it provides interesting hypotheses to explore about ESBL-E transmission between food-producing animals and humans in Madagascar and developing countries.

## Figures and Tables

**Table 1:** Prevalence of ESBL-E in livestock production farms of Reunion, Mayotte and Madagascar, 2016–2017.

Territory	N (Positive Farm)	ESBL-E Positive Farms	*p*-Value ^(1)^	*p*-Value ^(2)^
Reunion			<0.001	
Poultry	30 (21)	70.0% [53.3–86.7]	−	0.94
Pigs	30 (16)	53.3% [35.1–71.5]	−	<0.005
Beef cattle	54 (2)	03.7% [00.0–08.8]	−	<0.001
Mayotte			0.70	
Poultry	23 (17)	73.9% [55.6–92.2]	−	−
Beef cattle	19 (13)	68.4% [47.1–89.7]	−	−
Madagascar			0.16	
Poultry	30 (21)	70.0% [53.6–86.7]	−	−
Pigs	30 (26)	86.7% [74.3–99.1]	−	−
Beef cattle	30 (20)	66.7% [49.5–83.9]	−	−

N: total livestock commercial farms sampled. **^(1)^** *p*-value of Fisher test regarding occurrence between livestock production type in each territory. **^(2)^** *p*-value of Fisher test regarding occurrence in each livestock production type between each territory.

**Table 2:** Diversity of the ESBL-E species isolated in chromogenic agar from livestock production farms of Reunion, Mayotte and Madagascar, 2016–2017.

Bacterial Species		Reunion						Mayotte				Madagascar					
		Poultry		Pig		Cattle		Poultry		Cattle		Poultry		Pig		Cattle	
	N (% ESBL-E)	n	ESBL-E (%)	n	ESBL-E (%)	n	ESBL-E (%)	n	ESBL-E (%)	n	ESBL-E (%)	n	ESBL-E (%)	n	ESBL-E (%)	n	ESBL-E (%)
*Citrobacter freundii*	6 (100.0%)	-	-	2	2 (100.0%)	-	-	-	-	-	-	-	-	4	4 (100.0%)	-	-
*Escherichia coli*	307 (95.1%)	145	136 (93.8%) (93.8%)	45	40 (88.9%)	2	2 (100.0)	19	19 (100.0%)	17	17 (100.0%)	28	28 (100.0%) (100.0%)	29	28 (96.6%)	22	22 (100.0%)
*Escherichia hermannii*	2 (100.0%)	-	-	-	-	-	-	-		-	-	-	-	-	-	2	2 (100.0%)
*Enterobacter cloacae complex*	13 (92.3%)	1	1 (100.0%)	-	-	1	0 (00.0%)	1	1 (100.0%)	1	1 (100.0%)	1	1 (100.0%)	6	6 (100.0%)	2	2 (100.0%)
*Klebsiella pneumoniae*	11 (100.0%)	-	-	2	2 (100.0%)	-	-	-	-	-	-	2	2 (100.0%)	7	7 (100.0%)	-	-
*Morganella morganii*	2 (100.0%)	-	-	-	-	-	-	-	-	-	-	-	-	2	2 (100.0%)	-	-

**Table 3:** Antibiogram results of ESBL-E from livestock production farms of Reunion, Mayotte and Madagascar, 2016–2017.

	ETP			NA			OFL			GEN			AMK			SXT			TCN			
	S	I	R	S	I	R	S	I	R	S	I	R	S	I	R	S	I	R	S	I	R	ND
Reunion																						
Broiler																						
*E. coli* (N = 136)	136 (100.0%)	0 (00.0%)	0 (00.0%)	102 (75.0%)	5 (03.7%)	29 (21.3%)	131 (96.3%)	2 (01.5%)	3 (02.2%)	128 (94.1%)	0 (00.0%)	8 (05.9%)	134 (100.0%)	0 (00.0%)	0 (00.0%)	34 (25.0%)	0 (00.0%)	102 75.0%)	33 (24.3%)	0 (00.0%)	65 (47.8%)	38 (27.9%)
*E. cloacae* (N = 1)	1 (100.0%)	0 (00.0%)	0 (00.0%)	1 (100.0%)	0 (00.0%)	0 (00.0%)	1 (100.0%)	0 (00.0%)	0 (00.0%)	1 (100.0%)	0 (00.0%)	0 (00.0%)	1 (100.0%)	0 (00.0%)	0 (00.0%)	0 (00.0%)	0 (00.0%)	1 (100.0%)	1 (100.0%)	0 (00.0%)	0 (00.0%)	
Pig																						
*E. coli* (N = 40)	39 (97.5%)	1 (02.5%)	0 (00.0%)	29 (72.5%)	1 (02.5%)	10 (25.0%)	30 (75.0%)	0 (00.0%)	10 (25.0%)	35 (87.5%)	0 (00.0%)	5 (12.5%)	40 (100.0%)	0 (00.0%)	0 (00.0%)	5 (12.5%)	0 (00.0%)	35 (87.5%)	5 (12.5%)	1 (02.5%)	23 (57.5%)	11 (27.5%)
*C. freundii* (N = 2)	2 (100.0%)	0 (00.0%)	0 (00.0%)	2 (100.0%)	0 (00.0%)	0 (00.0%)	2 (100.0%)	0 (00.0%)	0 (00.0%)	2 (100.0%)	0 (00.0%)	0 (00.0%)	2 (100.0%)	0 (00.0%)	0 (00.0%)	2 (100.0%)	0 (00.0%)	0 (00.0%)	2 (100.0%)	0 (00.0%)	0 (00.0%)	
*K. pneumoniae* (N = 2)	1 (50.0%)	1 (50.0%)	0 (00.0%)	1 (50.0%)	0 (00.0%)	1 (50.0%)	1 (50.0%)	0 (00.0%)	1 (50.0%)	2 (100.0%)	0 (00.0%)	0 (00.0%)	2 (100.0%)	0 (00.0%)	0 (00.0%)	1 (50.0%)	2 (100.0%)	1 (50.0%)	1 (50.0%)	0 (00.0%)	1 (50.0%)	
Beef cattle																						
*E. coli* (N = 2)	2 (100.0%)	0 (00.0%)	0 (00.0%)	1 (50.0%)	0 (00.0%)	1 (50.0%)	1 (50.0%)	1 (50.0%)	0 (00.0%)	2 (100.0%)	0 (00.0%)	0 (00.0%)	2 (100.0%)	0 (00.0%)	0 (00.0%)	2 (100.0%)	0 (00.0%)	0 (00.0%)	0 (00.0%)	0 (00.0%)	2 (100.0%)	
Mayotte																						
Broiler																						
*E. coli* (N = 19)	19 (100.0%)	0 (00.0%)	0 (00.0%)	14 (73.7%)	4 (21.1%)	1 (05.3%)	19 (100.0%)	0 (00.0%)	0 (00.0%)	18 (94.7%)	0 (00.0%)	1 (05.3%)	19 (100.0%)	0 (00.0%)	0 (00.0%)	14 (73.7%)	0 (00.0%)	5 (26.3%)	3 (15.8%)	0 (00.0%)	16 (84.2%)	
*E. cloacae* (N = 1)	1 (100.0%)	0 (00.0%)	0 (00.0%)	0 (00.0%)	0 (00.0%)	1 (100.0%)	0 (00.0%)	1 (100.0%)	0 (00.0%)	0 (00.0%)	0 (00.0%)	1 (100.0%)	1 (100.0%)	0 (00.0%)	0 (00.0%)	0 (00.0%)	0 (00.0%)	1 (100.0%)	0 (00.0%)	0 (00.0%)	1 (100.0%)	
Beef cattle																						
*E. coli* (N = 16) *	16 (100.0%)	0 (00.0%)	0 (00.0%)	7 (43.8%)	5 (31.3%)	4 (25.0%)	14 (87.5%)	2 (12.5%)	0 (00.0%)	12 (75.0%)	0 (00.0%)	4 (25.0%)	16 (100.0%)	0 (00.0%)	0 (00.0%)	15 (93.8%)	0 (00.0%)	1 (06.3%)	12 (75.0%)	0 (00.0%)	4 (25.0%)	
*E. cloacae* (N = 1)	1 (100.0%)	0 (00.0%)	0 (00.0%)	0 (00.0%)	0 (00.0%)	1 (100.0%)	1 (100.0%)	0 (00.0%)	0 (00.0%)	1 (100.0%)	0 (00.0%)	0 (00.0%)	1 (100.0%)	0 (00.0%)	0 (00.0%)	1 (100.0%)	0 (00.0%)	0 (00.0%)	0 (00.0%)	1 (100.0%)	0 (00.0%)	
Madagascar																						
Broiler																						
*E. coli* (N = 28)	28 (100.0%)	0 (00.0%)	0 (00.0%)	13 (46.4%)	7 (25.0%)	8 (28.6%)	22 (78.6%)	3 (10.7%)	3 (10.7%)	27 (96.4%)	0 (00.0%)	1 (03.6%)	28 (100.0%)	0 (00.0%)	0 (00.0%)	27 (96.4%)	0 (00.0%)	1 (03.6%)	1 (03.6%)	1 (03.6%)	26 (92.9%)	
*E. cloacae* (N = 1)	1 (100.0%)	0 (00.0%)	0 (00.0%)	0 (00.0%)	0 (00.0%)	1 (100.0%)	0 (00.0%)	0 (00.0%)	1 (100.0%)	1 (100.0%)	0 (00.0%)	0 (00.0%)	1 (100.0%)	0 (00.0%)	0 (00.0%)	1 (100.0%)	0 (00.0%)	0 (00.0%)	0 (00.0%)	0 (00.0%)	1 (100.0%)	
*K. pneumoniae* (N = 2)	2 (100.0%)	0 (00.0%)	0 (00.0%)	0 (00.0%)	0 (00.0%)	1 (100.0%)	0 (00.0%)	0 (00.0%)	1 (100.0%)	0 (00.0%)	0 (00.0%)	1 (100.0%)	1 (100.0%)	0 (00.0%)	0 (00.0%)	0 (00.0%)	0 (00.0%)	2 (100.0%)	0 (00.0%)	0 (00.0%)	1 (100.0%)	
Pig																						
*E. coli* (N = 28)	28 (100.0%)	0 (00.0%)	0 (00.0%)	13 (46.4%)	8 (28.6%)	7 (25.0%)	20 (71.4%)	2 (07.1%)	6 (21.4%)	28 (100.0%)	0 (00.0%)	0 (00.0%)	28 (100.0%)	0 (00.0%)	0 (00.0%)	28 (100.0%)	0 (00.0%)	0 (00.0%)	7 (25.0%)	0 (00.0%)	21 (75.0%)	
*E. cloacae* (N = 6)	6 (100.0%)	0 (00.0%)	0 (00.0%)	2 (33.3%)	2 (33.3%)	2 (33.3%)	6 (100.0%)	0 (00.0%)	0 (00.0%)	4 (66.7%)	0 (00.0%)	2 (33.3%)	6 (100.0%)	0 (00.0%)	0 (00.0%)	4 (66.7%)	0 (00.0%)	2 (33.3%)	0 (00.0%)	0 (00.0%)	6 (100.0%)	
*C. freundii* (N = 4)	4 (100.0%)	0 (00.0%)	0 (00.0%)	0 (00.0%)	0 (00.0%)	4 (100.0%)	0 (00.0%)	0 (00.0%)	4 (100.0%)	1 (25.0%)	0 (00.0%)	3 (75.0%)	4 (100.0%)	0 (00.0%)	0 (00.0%)	1 (25.0%)	0 (00.0%)	3 (75.0%)	0 (00.0%)	0 (00.0%)	4 (100.0%)	
*M. morganii* (N = 6)	6 (100.0%)	0 (00.0%)	0 (00.0%)	0 (00.0%)	0 (00.0%)	6 (100.0%)	0 (00.0%)	0 (00.0%)	6 (100.0%)	0 (00.0%)	0 (00.0%)	6 (100.0%)	6 (100.0%)	0 (00.0%)	0 (00.0%)	0 (00.0%)	0 (00.0%)	6 (100.0%)	0 (00.0%)	0 (00.0%)	6 (100.0%)	
*K. pneumoniae* (N = 7)	7 (100.0%)	0 (00.0%)	0 (00.0%)	0 (00.0%)	3 (42.9%)	4 (57.1%)	5 (71.4%)	0 (00.0%)	2 (28.6%)	0 (00.0%)	0 (00.0%)	7 (100.0%)	7 (100.0%)	0 (00.0%)	0 (00.0%)	0 (00.0%)	0 (00.0%)	7 (100.0%)	0 (00.0%)	0 (00.0%)	7 (100.0%)	
Beef cattle																						
*E. coli* (N = 22)	22 (100.0%)	0 (00.0%)	0 (00.0%)	15 (68.2%)	3 (13.6%)	4 (18.2%)	18 (81.8%)	3 (13.6%)	1 (04.5%)	21 (95.5%)	0 (00.0%)	1 (04.5%)	22 (100.0%)	0 (00.0%)	0 (00.0%)	21 (95.5%)	0 (00.0%)	1 (04.5%)	11 (50.0%)	0 (00.0%)	11 (50.0%)	
*E. cloacae* (N = 2)	2 (100.0%)	0 (00.0%)	0 (00.0%)	2 (100.0%)	0 (00.0%)	0 (00.0%)	2 (100.0%)	0 (00.0%)	0 (00.0%)	2 (100.0%)	0 (00.0%)	0 (00.0%)	2 (100.0%)	0 (00.0%)	0 (00.0%)	2 (100.0%)	0 (00.0%)	0 (00.0%)	0 (00.0%)	0 (00.0%)	2 (100.0%)	
*E. hermannii* (N = 2)	2 (100.0%)	0 (00.0%)	0 (00.0%)	2 (100.0%)	0 (00.0%)	0 (00.0%)	2 (100.0%)	0 (00.0%)	0 (00.0%)	2 (100.0%)	0 (00.0%)	0 (00.0%)	2 (100.0%)	0 (00.0%)	0 (00.0%)	2 (100.0%)	0 (00.0%)	0 (00.0%)	0 (00.0%)	0 (00.0%)	2 (100.0%)	

ETP: ertapenem; NA: nalidixic acid; OFL: Ofloxacin; GEN: gentamicin; AMK: amikacin; SXT: trimethoprim/sulfamethoxazole; TCN: tetracyclin * One ESBL producing *E. coli* was lost at the laboratory. Antibiograms was performed on 16 of the 17 ESBL-E.

**Table 4:** ESBL genes identified in a subset of *E. coli* isolated from poultry, pig and beef cattle production farms in Reunion, Mayotte and Madagascar, 2016–2017.

Territory/	*E. coli* Tested	ESBL Genes Identified (%)							
Production Type		ND	CTX-M-1 Group				CTX-M-9 Group	SHV	TEM
Enzymes			CTX-M-1	CTX-M-3	CTX-M-15	CTX-M-32			
		**Reunion**																
Poultry	35	3	29 (90.6%)	-	-	-	-	1 (3.1%)	2 (6.3%)
Pigs	10	-	7 (70.0%)	1 (10.0%)	2 (20.0%)	-	-	-	-
Beef cattle	2	-	2 (100.0%)	-	-	-	-	-	-
**Mayotte**									
Poultry	10	1	7 (77.8%)	1 (11.1%)	1 (11.1%)	-	-	-	-
Beef cattle	10	3	1 (14.3%)	-	5 (71.4%)	1 (14.3%)	-	-	-
**Madagascar**									
Poultry	10	-	5 (50.0%)	-	2 (20.0%)	-	3 (30.0%)	-	-
Pigs	9 *	-	-	-	9 (100.0%)	-	-	-	-
Beef cattle	9 *	-	-	-	9 (100.0%)	-	-	-	-
TOTAL	95 (100.0%)	7 (7.4%)	51 (53.7%)	2 (2.1%)	28 (29.5%)	1 (1.1%)	3 (3.2%)	1 (1.1%)	2 (2.1%)

* Reunion and Madagascar only, (**a**) Intercept = 0.01376, null deviance: 99.832, model *d.f*. = 4; (**b**) Intercept = −2.7919, null deviance: 73.304, model *d.f*. = 3; (**c**) Intercept = 0.9959, null deviance: 132.027, model *d.f.* = 5.

**Table 5:** Bivariate explanatory factors of ESBL-E occurrence in livestock from Reunion, Mayotte and Madagascar, 2016–2017.

Country	Livestock	Variable	OR, IC95%	*p*-Value
Reunion	Broiler	Premises building constructed > 1999	12.72 [1.25–671.77]	0.01
	Pigs	Change clothes before entering house/pen	6.52 [0.92–80.50]	0.05
		Change shoes/boots before entering house/pen	13.62 [1.35–716.37]	0.01
		Rodent control by a company	0.11 [0.01–0.75]	0.01
		Lightning in the building	0.18 [0.01–2.13]	0.04
		Two disinfections between two consecutive batches	0 [0–0.92]	0.04
	Beef cattle cows	−	−	−
Madagascar	Broiler	Chicks produced in the farm	0 [0.00–0.91]	0.02
	Pigs	Use of antibiotic for prophylaxis	0.09 [0.00–1.36]	0.05
	Beef cattle	Clearing space around the building	0 [0.00–0.94]	0.03
		Clean condition around the farm	0 [0–1.94]	0.003
Mayotte	Broiler	Distance from another poultry farm (>500 m)	13.39 [0.79–883.37]	0.04
	Beef cattle	−	−	−

**Table 6:** Best model explaining ESBL-E occurrence in poultry, pig and cow production (including all territories), 2016−2017.

Dependent Variables	Independent Variables	Adj. OR (CI95%)	*p*-Value	AIC
Broiler occurrence (a)	Distance elev oth species >500 m	3.18 (0.65–15.56)	0.15	93.68
	Distance elev oth species <500 m	0.99 (0.26–4.39)	0.99	
	Foot bath at room entrance	5.89 (0.61–57.17)	0.13	
	Water quality control	0.12 (0.02–0.82)	0.03	
	Water storage tank	2.58 (0.85–7.79)	0.09	
Pig occurrence * (b)	Farmers visits	14.15 (1.17–171.35)	0.04	65.09
	Soak the floor	22.34 (1.51–330.98)	0.02	
	Detergent use for cleaning	0.12 (0.02–0.75)	0.02	
	Antibiotic use recently (<1 year)	8.82 (1.09–71.4)	0.04	
Beef cattle occurrence (c)	Livestock size > 25	0.07 (0.02–0.28)	<0.001	83.53
	Antibiotic drug use recently (<1 year)	3.94 (1.04–14.98)	0.04	
	Disinfestation	0.19 (0.04–0.91)	0.04	
	Clearing space around the building	0.22 (0.04–1.29)	0.09	
	Water storage tank	0.38 (0.11–1.35)	0.14	
	Pet presence	6.87 (1.13–41.67)	0.04	

* Reunion and Madagascar only, (**a**) Intercept = 0.01376, null deviance: 99.832, model *d.f*. = 4; (**b**) Intercept = −2.7919, null deviance: 73.304, model *d.f*. = 3; (**c**) Intercept = 0.9959, null deviance: 132.027, model *d.f.* = 5.

## Supplementary Materials

The following are available online at http://www.mdpi.com/2306-7381/5/1/22/s1, Table S1: Questionnaire.
