# Supplementary material for: Risk Factors of Extended-Spectrum β-Lactamase Producing Enterobacteriaceae Occurrence in Farms in Reunion, Madagascar and Mayotte Islands, 2016–2017
Source: Vet Sci. 2018 Feb 23;5(1):22. doi: 10.3390/vetsci5010022 (PMC5876575; doi:10.3390/vetsci5010022)
Supplement: Supplementary file 1 [file vetsci-05-00022-s001.pdf]

Supplementary Materials: Risk factors of Extended-Spectrum β-Lactamase producing Enterobacteriaceae occurrence in farms in Reunion, Madagascar and Mayotte Islands, 2016–2017

Noellie Gay *, Alexandre Leclaire, Morgane Laval, Guillaume Miltgen, Maël Jégo, Ramin Stéphane, Julien Jaubert, Olivier Belmonte and Eric Cardinale

Name and code of the farmer : .... Territory : ....
Date : / / 16

Animal species	Herd size

Building

Presence of a water source near	River	Pond	Purification station or other	None
If yes, distance from the farm	<100m	>100m		
Closed building	Yes	No		
Concrete access to building	Yes	No		
More than one building	Yes	No		
if Yes<br>Building number in the exploitation	1	2	≥2	
Age of the livestock exploitation				
Other livestock species in the farm	No	Cow	Pig	Poultry	Other :……..
Distance from another farm (same species)	< 500 m	> 500 m		
Distance from another farm (other species raised)	< 500 m	> 500 m		
If Yes<br>Type of species	Cow	Pig	Other :	
Vegetable production on the farm	Yes	No	Type :	
Clean condition around the farm	Yes	No		

Hygiene and biosecurity

**Hygiene measure**				
Presence of a vestibule at building entrance	Yes	No		
Boot bath at building entrance	Yes	No		
Presence of a lavabo closeby	Yes	No	Showers ?	
Change of work clothes at building entrance	Yes	No		
Change of shoes/boots at building entrance	Yes	No		
Clothes and shoes	Durty	Clean		
Entrance frequency in the building by day	≤3	4	>4	
Other farmers visiting the exploitation	Yes	No		
Share equipement with an other exploitation	Yes	No		
Share workers with an other exploitation	Yes	No		
Boot bath at building entrance	Yes	No		
Respect of biosecurity measures	Yes	No		
**Rendering**				
Management of dead livestock	Consumption	Buried	Burned	Thrown
**Water**				
Type of water used in the exploitation	Public	River	Rain<br>Other: well, etc.	
Water storage presence	Yes	No		
Potable quality control	Yes	No		
Water filtration	Yes	No		
Water treatment	Yes	No		
Water quality control	Yes	No		
Cleaning of water pipes	Yes	No	No pipes	
**Vector control**				
Rodent presence	Yes	No		
Rodent control	None	By farmer	By a society	
If yes Type of control	Poison	Traps	Other :	
Clearing around the farm	Yes	No		
Desinfestation	Yes	No		
Pet presence in the farm	Yes	No	Species :	
Could bird enter the building	Yes	No		
Presence of flies	Elevated	Moderate		
**Quarantine**				
If buying breeding stock				
Quarantine before entering the stock	Yes	No		
Quarantaine in a different building	Yes (>50m)	Yes (10-50m)	No	

All in all out practice	Yes	No			
Cleaning before next batch	Yes	No			
**Effluents**					
Manure storage on exploitation	Yes		No		
Spreading of the manure on the exploitation	Yes		No		
Si Yes<br>Distance from the farm					
Spreading of the manure on another farm	Yes		No		
**Cleaning and disinfection**					
Scraping manure	Yes	No			
Soaking surface	Yes	No			
Water use for cleaning	Public	River	rain	Other :	
Detergent use for cleaning	Yes	No			
Cleanied surfaces	Ground	Ceiling	Wall		
		Through			
Application	Bucket	Sprayer	Lather	Other :	
Pickling	Yes	No			
Lightning in the building	Good	Moderate	Bad		
Crawlspace	Yes	No			
Two desinfectations	Yes	No			
Clean condition around the farm	Clean	Durty			

B – Antibiotic use :

- **Motivation** : Therapeutic Prophylactic

Last antibiotic treatment : <1 year >1 year

Antibiotic use in : previous livestock sampled one
